# Importance of muldisciplinary management of giant mediastinal sarcoma: A case report with phrenic nerve reconstruction 

**DOI:** 10.1111/1759-7714.13452

**Published:** 2020-04-23

**Authors:** Luca Frasca, Filippo Longo, Giovanni Tacchi, Francesco Stilo, Anna Zito, Beniamino Brunetti, Massimiliano Depalma, Pierfilippo Crucitti

**Affiliations:** ^1^ Department of Thoracic Surgery Campus Bio‐Medico University of Rome Rome Italy; ^2^ Department of Vascular Surgery Campus Bio‐Medico University of Rome Rome Italy; ^3^ Department of Geriatrics Campus Bio‐Medico University of Rome Rome Italy; ^4^ Department of Reconstructive and Aesthetic Surgery Campus Bio‐Medico University of Rome Rome Italy

**Keywords:** Diaphragm paralysis, mediastinal mass, multidiscipline, nerve reconstruction, phrenic nerve, sarcoma

## Abstract

Primary synovial sarcoma is a soft tissue tumor which originates from synovial‐like undeveloped mesenchymal structures. Here, we report the case of a giant mediastinal sarcoma in a 41‐year‐old female patient. After diagnosis, she underwent neoadjuvant chemotherapy. Due to its low efficacy, we collegially decided to undergo cytoreductive debulking surgery. The mass invaded the phrenic nerve bilaterally and its excision caused a severe lesion of the left nerve and a partial impairment of the right one. Thus, plastic surgeons decided to reconstruct the right phrenic nerve employing the contralateral remaining fibers.

The invasiveness of this tumor, its difficult removal, histological profile and the peculiar technique to preserve diaphragmatic function classify this case as very rare. The therapeutic strategy was based on interdisciplinary teamwork which comprised several specialists' opinions. Our strategy allowed us to pursue the challenging objective to give a young woman with a severe diagnosis the best possible chance of achieving a good quality of life.

To the best of our knowledge, this phrenic nerve reconstructive technique is very rare and has not previously been reported in the literature. The report emphasizes that it is possible to deal with an apparently impossible case through a collaborative approach involving several different medical specialist professionals.

## Introduction

Primary synovial sarcoma is an uncommon soft‐tissue tumor. Synovial sarcomas originate from undeveloped mesenchymal structures, which bear a resemblance to synovial tissue.^1^, ^2^, ^3^ When occurring in the mediastinum, they can involve various structures such as nerves.^4^, ^5^, ^6^ Thus, debulking surgery can be followed by reconstructive techniques.^7^ The peculiar neurosurgical strategy we performed consisted of the transplantation of contralateral nerve fibers. Here, we report the case of a female patient with a huge synovial sarcoma, located in the mediastinum.^8^, ^9^, ^10^ This report has been written in accordance with the Surgical Case Report (SCARE) criteria.

### Case report

**Figure 1:**
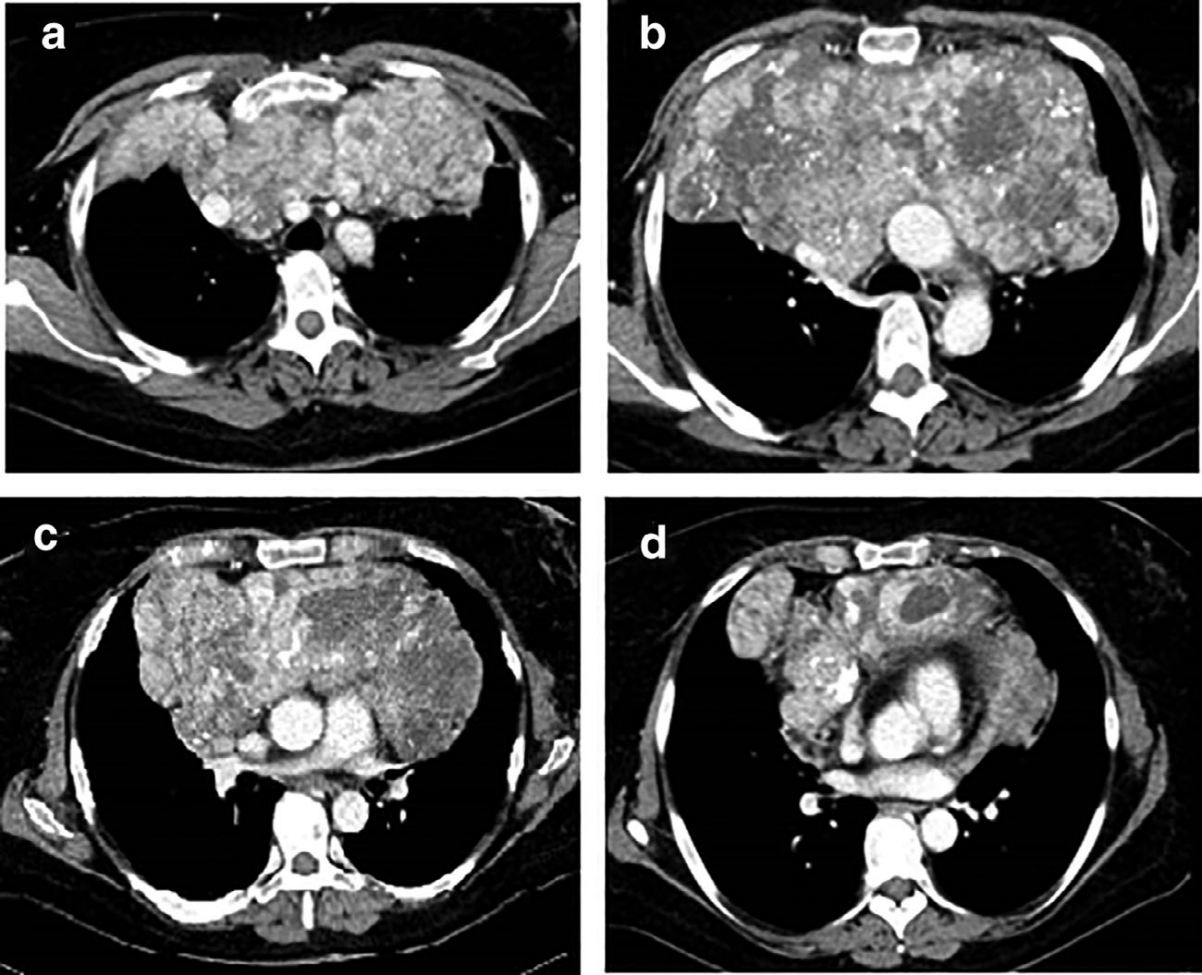
Preoperative chest axial view computed tomography (CT) scan of the heterogeneous mass.

**Figure 2:**
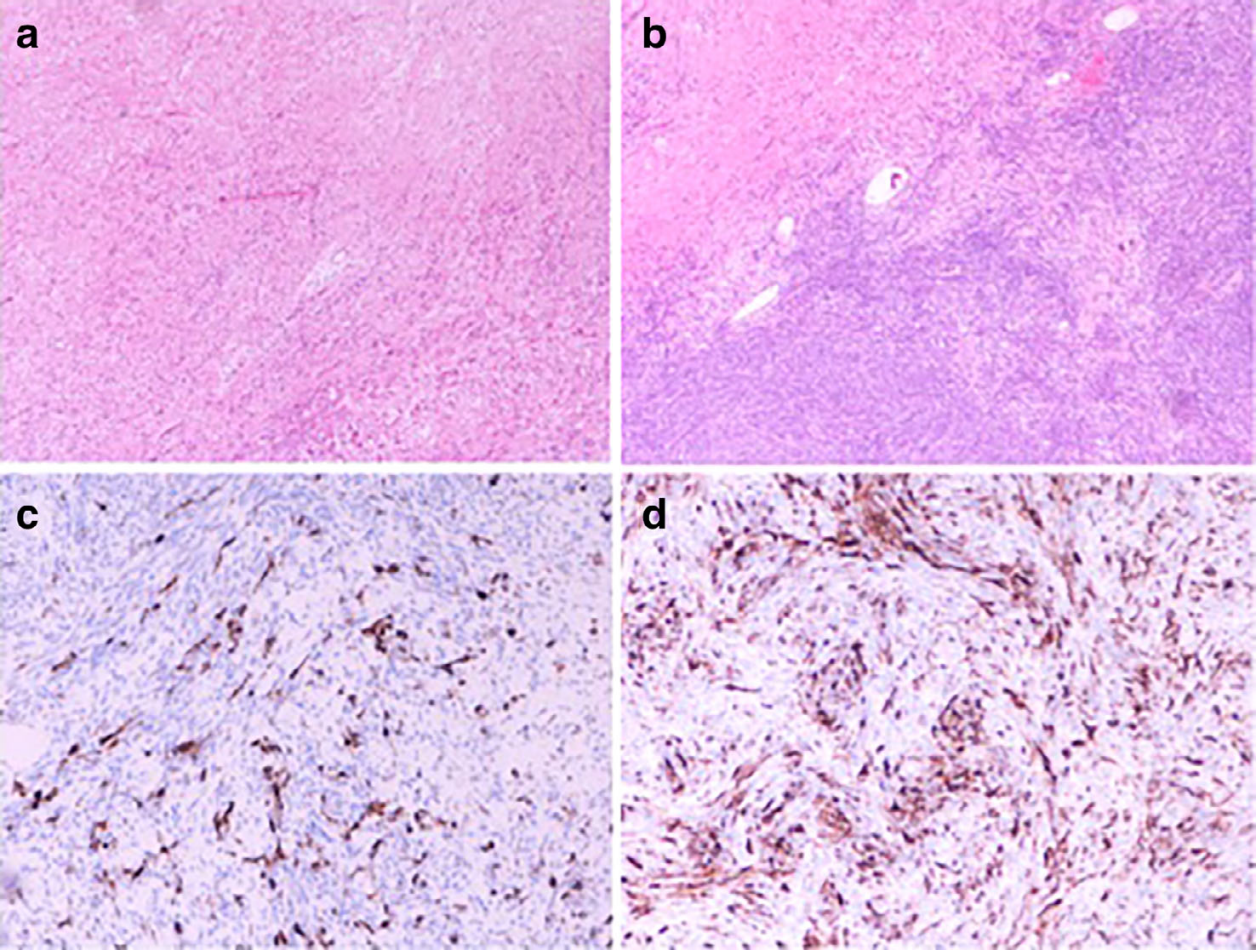
Synovial sarcoma: Microphotograph fixed with Hematoxylin and eosin (**a**,**b**) (4X), Microphotograph with Immunohistochemistry (**c,d**).

A 41‐year‐old patient presented to the Emergency Unit with severe dyspnea. She was an ex‐athlete, hypertensive and a current 23 pack‐year smoker. A contrast computed tomography (CT) scan of the chest was performed which revealed a large anterior mediastinal mass with heterogenous enhancement (Fig 1). The mass was adherent to the superior vena cava (SVC), pulmonary vessels, aortic arch and pericardium. In addition, it compressed the trachea and principal bronchial branches. Histological examination based on specimens obtained via anterior mediastinoscopy underlined the presence of a monophasic synovial sarcoma evidenced by monomorphic spindle cells, organized into bundles, with increased ratio of nuclear material to cytoplasm (Fig 2). Cells showed indistinct margins and frequent dystrophic calcifications (Fig 2a,b).

Immunohistochemical technique was positive for cytokeratine AE1‐AE3, epthielial membrane antigen, andtransducin‐like enhancer 1 (Fig 2c,d).^11^

The patient commenced six cycles of chemotherapy based on Epirubicin 60 mg/Ifosfamide 4500 mg for four months. After one month following chemotherapy, the control CT scan did not demonstrate any significant decrease in the dimension of the mass. In accordance with a multidisciplinary team (MDT) comprising oncologists, thoracic, vascular and plastic surgeons, pulmonologists, radiologists, anesthetists and pathologists, it was agreed that the patient was a candidate for a cytoreductive debulking intervention.

A right hemi‐clamshell incision was performed. A left anterolateral thoracotomy was provided for better control of the mediastinal vascular structures. The tumor encased the right phrenic nerve for one third of the course, and left phrenic nerve for two thirds of the course. The mass was firstly removed from the costal surface in order to access the mediastinum from the SVC side. After having clamping the SVC and its tributaries, we incised the SVC through its course in the direction of the right atrium: it was infiltration by the tumor. Therefore, we removed this portion of the SVC anterior wall with a neoplastic thrombus. We then reconstructed the SVC by placing a patch.

Thereafter, the neoplasia was entirely removed. According to the MDT treatment plan, plastic surgeons subsequently reassembled the phrenic nerve fibers. Right phrenic nerve residuals were, after resection of the tumor, more represented than left ones. Consequently, the right diaphragmatic nerve was reconstructed with a 10 cm orthotopic graft (outside of the tumoral area) belonging to the residual left phrenic nerve. To perform an end‐to‐end coaptation, an epineural microsurgical 8–0 prolene suture was used and secured with 0.5 mL of fibrin glue. Finally, we fixed a goretex‐type prosthesis to the sternum (medially) and to the second rib (laterally). The thoracic wall was stabilized by placing a titanium bar to the anterior arch of both the second ribs.

**Figure 3:**
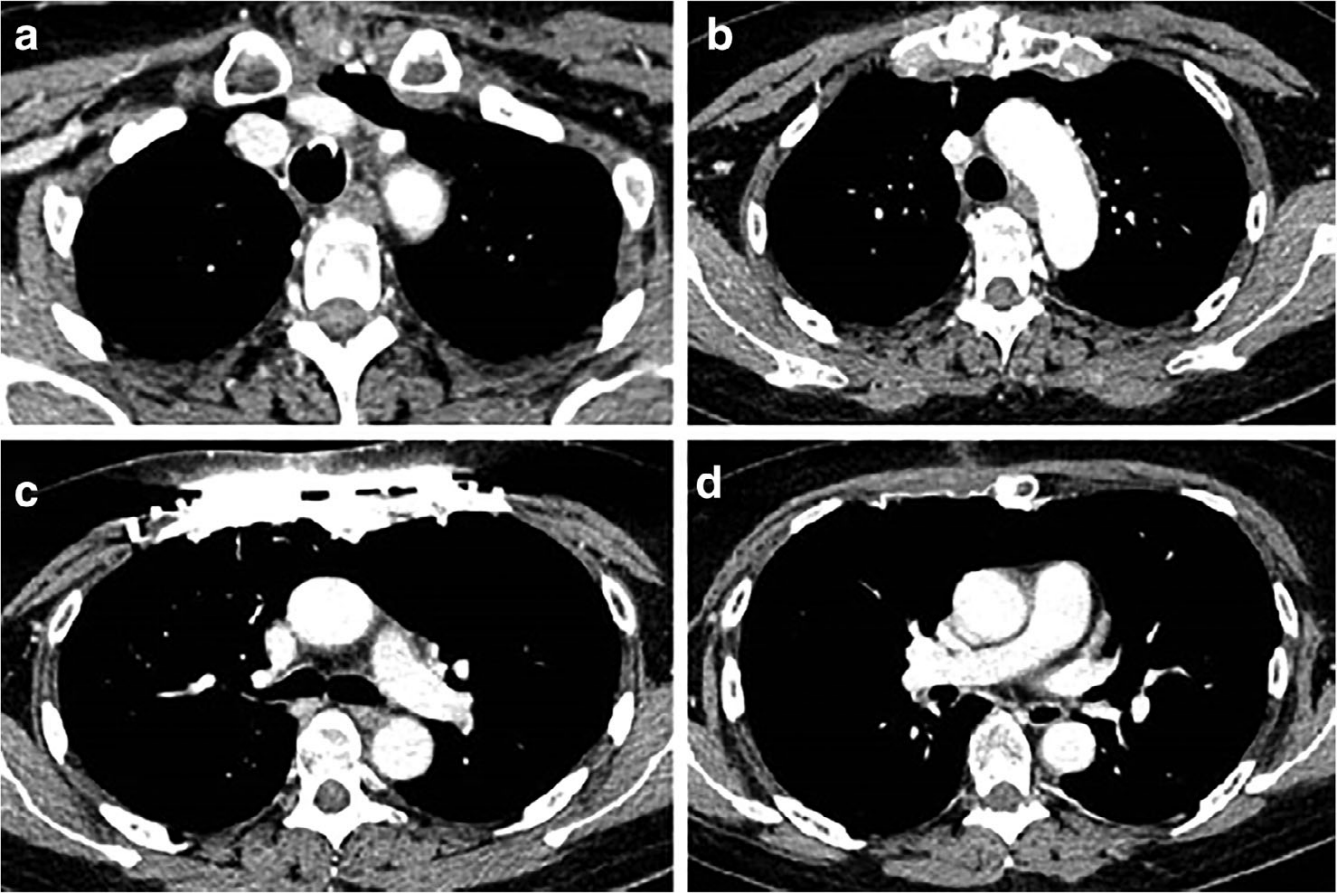
Postoperative axial view chest CT.

The orotracheal tube was removed 48 hours after the procedure and the patient was tracheostomized and mechanically ventilated in order to maintain initial paralysis of the diaphragm. After two weeks, the first postoperative CT scan was executed (Fig 3). In the subsequent weeks, with the aid of regular exercise of auxiliary respiration muscles, there was an improvement in respiratory function evidenced by serial arterial blood gases (ABG) (Table 1). During the third month follow‐up, a thoracic M‐mode scan was carried out which evaluated diaphragmatic movement with regard to maximal inspiration and expiration. The three diaphragmatic excursion measurements were 6.6 cm, 7.0 cm and 7.5 cm, respectively. Realized values were within normal limits and underlined a satisfactory restoration of respiratory capacity. Four months after the procedure, the patient underwent an overnight polysomnography, which underlined the absence of sleep apnea or significant hypoxemia and suggested that it might be possible to remove her tracheostomy. At the beginning of the fifth month, her tracheostomy was removed.

**Table 1:** Results of respiratory function evidenced by serial arterial blood gases (ABG)

	Duration after surgery			
Parameters	2 weeks	6 weeks	8 weeks	16 weeks
Ph	7.43	7.46	7.43	7.43
PaCO_2_, mmHg	46	35	39	40
PaO_2_, mmHg	79	80	83	89
Sat O_2_, %	98.5	99	93,2	99.5
HCO_3‐_ mmol/L	29.2	25.9	25.9	26.5
Lac, mEq/L	9	8	8	6

## Discussion

According to Schoeller *et al*. prompt microsurgical reconstruction might be the optimal treatment for diaphragmatic dysfunction caused by tumor infiltration of the phrenic nerve once curative resection of the tumor has been performed.^12^ Follow‐up of patients on which this type of procedure has been carried out have demonstrated optimal results.^13^, ^14^, ^15^, ^16^, ^17^ To the best of our knowledge, there have been no reports in the literature which have focused on the technique we performed here, even though some teams have proposed other approaches such as neurotization of the phrenic nerve with the trapezius branch of the ipsilateral spinal accessory nerve, or reconstruction of the phrenic nerve employing fibers of sural nerve.^18^ In the light of chemotherapeutic failure in this patient, a surgical option was derived from a MDT decision, and this approach enabled removal of the tumor which offered a better life expectancy, as well as achieving spontaneous breathing effort.

## Disclosure

The authors declare there are no conflicts of interest.
